# Rapid SARS-CoV-2 antigen detection assay in comparison with real-time RT-PCR assay for laboratory diagnosis of COVID-19 in Thailand

**DOI:** 10.1186/s12985-020-01452-5

**Published:** 2020-11-13

**Authors:** Chutikarn Chaimayo, Bualan Kaewnaphan, Nattaya Tanlieng, Niracha Athipanyasilp, Rujipas Sirijatuphat, Methee Chayakulkeeree, Nasikarn Angkasekwinai, Ruengpung Sutthent, Nattawut Puangpunngam, Theerawoot Tharmviboonsri, Orawan Pongraweewan, Suebwong Chuthapisith, Yongyut Sirivatanauksorn, Wannee Kantakamalakul, Navin Horthongkham

**Affiliations:** 1 Department of Microbiology, Faculty of Medicine Siriraj Hospital, Mahidol University, Bangkok, Thailand; 2 Department of Medicine, Faculty of Medicine Siriraj Hospital, Mahidol University, Bangkok, Thailand; 3 Department of Surgery, Faculty of Medicine Siriraj Hospital, Mahidol University, Bangkok, Thailand; 4 Department of Orthopaedic Surgery, Faculty of Medicine Siriraj Hospital, Mahidol University, Bangkok, Thailand; 5 Department of Anesthesiology, Faculty of Medicine Siriraj Hospital, Mahidol University, Bangkok, Thailand

**Keywords:** COVID-19, SARS-CoV-2, Rapid antigen, RT-PCR, Thailand

## Abstract

**Background:**

The Coronavirus disease 2019 (COVID-19) pandemic continues to spread across the world. Hence, there is an urgent need for rapid, simple, and accurate tests to diagnose severe acute respiratory syndrome coronavirus 2 (SARS-CoV-2) infection. Performance characteristics of the rapid SARS-CoV-2 antigen detection test should be evaluated and compared with the gold standard real-time reverse transcription-polymerase chain reaction (RT-PCR) test for diagnosis of COVID-19 cases.

**Methods:**

The rapid SARS-CoV-2 antigen detection test, Standard™ Q COVID-19 Ag kit (SD Biosensor®, Republic of Korea), was compared with the real-time RT-PCR test, Allplex™ 2019-nCoV Assay (Seegene®, Korea) for detection of SARS-CoV-2 in respiratory specimens. Four hundred fifty-four respiratory samples (mainly nasopharyngeal and throat swabs) were obtained from COVID-19 suspected cases and contact individuals, including pre-operative patients at Siriraj Hospital, Bangkok, Thailand during March–May 2020.

**Results:**

Of 454 respiratory samples, 60 (13.2%) were positive, and 394 (86.8%) were negative for SARS-CoV-2 RNA by real-time RT-PCR assay. The duration from onset to laboratory test in COVID-19 suspected cases and contact individuals ranged from 0 to 14 days with a median of 3 days. The rapid SARS-CoV-2 antigen detection test’s sensitivity and specificity were 98.33% (95% CI, 91.06–99.96%) and 98.73% (95% CI, 97.06–99.59%), respectively. One false negative test result was from a sample with a high real-time RT-PCR cycle threshold (Ct), while five false positive test results were from specimens of pre-operative patients.

**Conclusions:**

The rapid assay for SARS-CoV-2 antigen detection showed comparable sensitivity and specificity with the real-time RT-PCR assay. Thus, there is a potential use of this rapid and simple SARS-CoV-2 antigen detection test as a screening assay.

## Background

The Coronavirus disease 2019 (COVID-19) pandemic caused by the severe acute respiratory syndrome coronavirus 2 (SARS-CoV-2) has spread worldwide since its first recorded case in the city of Wuhan, China in December 2019. According to the COVID-19 Dashboard on August 31st, 2020 by the Center for Systems Science and Engineering (CSSE) at Johns Hopkins University, over 25 million people in more than 200 countries have been infected and killed more than 840,000 [1–3]. It is expected that these numbers continue to rise, especially in populous countries such as the United States, Brazil, and India. In Thailand, the first documented cases of COVID-19 were two Chinese tourists arriving from the city of Wuhan on January 8th and 13th, 2020, respectively. As of August 31st, 2020, there have been 3,412 confirmed COVID-19 cases with 58 deaths; 2,444 cases were from local transmission [4, 5]. The Thai government mandated a 14-day State Quarantine for all travelers entering Thailand from abroad. Since May 26th, 2020, no new local transmission cases were documented; new confirmed COVID-19 cases were people who have tested positive while in State Quarantine after returning from abroad [5]. SARS-CoV-2 infection causes asymptomatic and mild diseases more than severe pneumonia. Severe cases may develop acute respiratory distress syndrome (ARDS) and death with an average mortality rate of 6% (range 1–14.4%) [1, 3, 6].

The real-time reverse transcription-polymerase chain reaction (RT-PCR) assay, which is the current standard test for laboratory diagnosis of SARS-CoV-2 infection, requires at least four hours of operation performed by skilled technicians. Therefore, rapid and accurate tests for SARS-CoV-2 screening are essential to expedite disease prevention and control, as well as screening during pre-operative management for invasive procedures [7–9]. Lateral flow immunoassays using monoclonal anti-SARS-CoV-2 antibodies, which target SARS-CoV-2 antigens, can be the complementary screening tests if their accuracy were comparable to that of the real-time RT-PCR assays [10–13].

Here, we evaluated a rapid SARS-CoV-2 antigen detection test, Standard™ Q COVID-19 Ag kit (SD Biosensor®, Republic of Korea) using 454 respiratory specimens. The performance of this lateral flow immunoassay was compared with the SARS-CoV-2 RT-PCR for viral gene detection assay, Allplex™ 2019-nCoV Assay (Seegene®, Korea). This evaluation is critical before the implementation of the rapid antigen test for screening of SARS-CoV-2 infected individuals.

## Methods

### Ethical issues

This study was approved by the Institutional Review Board of the Faculty of Medicine Siriraj Hospital, Mahidol University (SIRB protocol 463/2563(IRB4); COA: Si 503/2020).

### Clinical specimens

Respiratory samples, mainly nasopharyngeal and throat swabs, were collected from 454 suspected COVID-19 cases, including pre-operative patients at Siriraj Hospital, Mahidol University, Bangkok, Thailand, from March to May 2020. Samples were mixed in 2 mL of viral transport media (VTM), consisting of Hanks’ balanced salt, 0.4% fetal bovine serum, HEPES, antibiotic and antifungal agents. Samples were transported at 2–8 °C to the Microbiology laboratory, Siriraj Hospital, for processing within a few hours. All specimens were processed in biosafety level-3 (BSL-3) and biosafety level-2 enhanced (BSL-2 +) facilities with full personal protective equipment.

### Viral RNA Extraction

MagLEAD 12gC automated extraction platform (Precision System Science, Chiba, Japan) was used to extract SARS-CoV-2 RNAs from 200 µL of nasopharyngeal and throat swabs. Extraction was performed according to the manufacturer’s instructions. Viral RNA was eluted with 100 µL buffer and used for RT-PCR assay.

### SARS-CoV-2 RNA detection using real-time RT-PCR

Allplex™ 2019-nCoV Assay (Seegene, Korea), which targets envelope gene (*E*) of *Sarbecovirus*, and RNA-dependent RNA polymerase (*RdRp*) and nucleocapsid (*N*) genes of SARS-CoV-2, was used for SARS-CoV-2 RNA detection according to the manufacturer’s instructions. Briefly, 8 μL of extracted RNA was added to 5 μL of 5X Real-time One-step Buffer, 5 μL of 2019-nCoV MuDT Oligo Mix (2019-nCoV-MOM), 2 μL of Real-time One-step Enzyme, and 5 μL of RNase free water. The CFX-96 real-time thermal cycler (Bio-Rad Laboratories, Inc., Hercules, CA, USA) was used for amplification. The conditions consisted of 1 cycle of 20 min at 50 °C, 1 min at 95 °C and followed by 45 cycles of 15 s at 94 °C, 30 s at 58 °C. The result was analysed using Seegene Viewer (Seegene, Korea), in which a cycle threshold value (Ct-value) < 40 for all three target genes was defined as a positive result.

### Rapid SARS-CoV-2 antigen detection assay

Standard Q COVID-19 Ag test (SD Biosensor®, Chuncheongbuk-do, Republic of Korea) is a rapid chromatographic immunoassay for the detection of SARS-CoV-2 nucleocapsid (N) antigen in respiratory specimens. This rapid antigen test device has two pre-coated lines on the result window: control (C) and test (T) lines. The control (C) region is coated with mouse monoclonal anti-chicken Igγ antibody; the test (T) region is coated with mouse monoclonal anti-SARS-CoV-2 antibody against SARS-CoV-2 N antigen. Detectors for SARS-CoV-2 N antigen presented in the specimen are mouse monoclonal anti-SARS-CoV-2 antibody conjugated with color particles. The antigen–antibody color particle complex migrates via capillary force and is captured by the mouse monoclonal anti-SARS-CoV-2 antibody coated on the test (T) region. The colored test (T) line’s intensity depends on the amount of SARS-CoV-2 N antigen presented in the sample.

This rapid Ag test kit was used for the detection of SARS-CoV-2 antigen in respiratory samples in this study. Specimens were processed in biosafety level-3 (BSL-3) and biosafety level-2 enhanced (BSL-2 +) facilities. Five to ten glass beads were added to the samples in VTM tubes. For highly viscous samples, additional VTM was added to reduce the viscosity. Specimens were mixed using a vortex mixer to disrupt thick mucus. The 200 μL of each nasopharyngeal and throat swab specimen was added to the extraction buffer provided in the kit. The filter nozzle cap was pressed tightly onto the extraction tube. Three drops of the extracted sample were applied on a test device, and the test result was read in 15–30 min. For positive COVID-19 antigen result, two colored lines of control (C) and test (T) lines were presented.

### Statistical analysis

Descriptive statistics were used to describe general information of patients. Continuous data were presented in mean, standard deviation (SD), median, and range. Categorical data were presented in numbers, percentages, and 95% confidence interval (95% CI). Sensitivity, specificity, positive predictive value (PPV), negative predictive value (NPV) were calculated using an online statistical tool [14].

## Results

### Characteristics of Thai COVID-19 cases

Suspected COVID-19 cases and contact individuals were laboratory-confirmed by the gold-standard RT-PCR assay as a national guideline for laboratory diagnosis of COVID-19 [15]. A total of 454 respiratory samples, including 447 nasopharyngeal (NP) and throat swabs, four endotracheal aspirates (tracheal suctions), and three sputum samples, were collected from suspected COVID-19 cases and pre-operative patients at Siriraj Hospital from March to May 2020. These respiratory samples were collected from subjects with the following conditions: (1) asymptomatic and upper respiratory tract infection individuals who had contacted with confirmed cases or were from COVID-19 risk areas, (2) clusters with acute respiratory infections, (3) unknown causative agents of pneumonia, (4) travelers screened at a port of entry and in quarantine places, and (5) pre-operative patients. Of the samples tested for COVID-19 (n = 454) by real-time RT-PCR assay, Allplex™ 2019-nCoV Assay, 13.2% (n = 60) were positive, while 86.8% (n = 394) were negative for SARS-CoV-2 RNA, as shown in Additional file 1: Supplementary Table S1, Additional file 2: Table S2.

The median age of Thai COVID-19 cases (n = 60) was 38.5 years (range 21–72). Male patients were found to be 60% of the infected cases (n = 36). Of the total COVID-19 cases, 75% (n = 45) of patients had direct contact with a variety of confirmed cases in Thailand, such as family members and friends (30%; n = 18), people from karaoke bars and pubs (23.3%; n = 14), people from boxing stadiums (18.3%; n = 11), taxi drivers (1.7%; n = 1), and peers in workplaces (1.7%; n = 1), as shown in Table 1. Most patients showed signs and symptoms of upper respiratory tract infections (61.7%; n = 37). Around 8.3% (n = 5) of COVID-19 cases were presented with pneumonia and were admitted to an intensive care unit (ICU). The median time from onset to laboratory tests for SARS-CoV-2 infection (both RT-PCR and rapid antigen detection assays) was three days (range 0–14), as shown in Table 1 and Additional file 1: Supplementary Table S1.

**Table 1:** Characteristics of COVID-19 Thai cases

Characteristics	Results
Number of COVID-19 cases	n = 60
Age (years)	
Median (range)	38.5 (range 21–72)
Gender	
Male	n = 36 (60%)
Risk factors	
Confirmed case contact	n = 45 (75%): boxing stadiums (n = 11), karaoke bars&pubs (n = 14), workplaces (n = 1), taxi drivers (n = 1), family members and friends (n = 18)
Foreign contact	n = 8 (13.3%): UK (n = 5), China (n = 1), India (n = 1), Cambodia (n = 1)
Public area contact	n = 2 (3.3%): public market (n = 1), domestic travel (n = 1)
Unspecified	n = 5 (8.3%)
Diagnoses	
Asymptomatic	n = 3 (5.0%)
URI	n = 37 (61.7%)
Fever	n = 11 (18.3%)
Pneumonia	n = 5 (8.3%)
Unspecified	n = 4 (6.7%)
Time from onset to laboratory test (days)	
Median (range)	3 (range 0–14)
Results of RT-PCR assay	
*Ct-value of E*	
Mean ± SD (min, max)	22.79 ± 6.69 (min 10.49, max 35.02)
*Ct-value of RdRp*	
Mean ± SD (min, max)	24.73 ± 6.55 (min 13.41, max 39.20)
*Ct-value of N*	
Mean ± SD (min, max)	26.09 ± 6.47 (min 12.07, max 37.17)
Results of rapid antigen detection assay	
Positive	59/60 (98.33%)
Negative	1/60 (1.67%)

### Real-time RT-PCR and SARS-CoV-2 antigen assays

Real-time RT-PCR (Allplex™ 2019-nCoV Assay), which targets *E* of *Sarbecovirus*, and *RdRp* and *N* genes of SARS-CoV-2, was used for SARS-CoV-2 RNA detection. The average cycle threshold (Ct) values in COVID-19 positive cases were 22.79 ± 6.69 (min 10.49, max 35.02) for *E* gene, 24.73 ± 6.55 (min 13.41, max 39.20) for *RdRp* gene, and 26.09 ± 6.47 (min 12.07, max 37.17) for *N* gene, as shown in Table 1 and Additional file 1: Supplementary Table S1. The negative RT-PCR results were defined as having a Ct-value higher than 40 for all three target genes (*E*, *RdRp*, *N*).

**Fig. 1:**
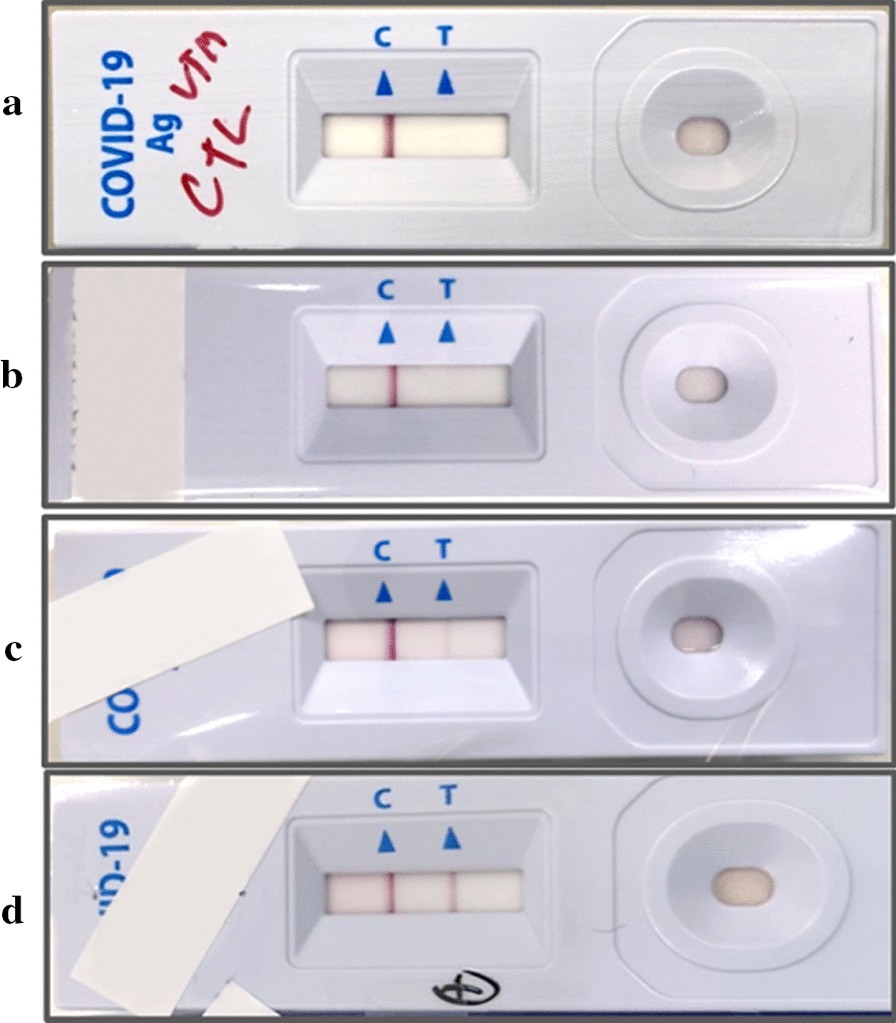
Interpretation of rapid SARS-CoV-2 antigen detection assay (Standard Q COVID-19 Ag Test). Demonstration of **a** a test strip for viral transport media control, **b** a test strip, which was interpreted as negative SARS-CoV-2 antigen, **c** a test strip, which was interpreted as (weakly) positive SARS-CoV-2 antigen, and **d** a test strip, which was interpreted as positive SARS-CoV-2 antigen. The results were interpreted as positive when both control (**c**) and SARS-CoV-2 antigen (T) lines appeared within 30 min

We evaluated the performance characteristics of SARS-CoV-2 antigen detection (Standard Q COVID-19 Ag test). The results were interpreted as positive when both control (C) and SARS-CoV-2 antigen (T) lines appeared within 30 min, as shown in Fig. 1. Comparing SARS-CoV-2 antigen detection to RNA detection by RT-PCR assay, the sensitivity and specificity of rapid SARS-CoV-2 antigen detection to identify COVID-19 were 98.33% (59/60; 95%CI, 91.06–99.96%) and 98.73% (389/394; 95%CI, 97.06–99.59%), respectively, as shown in Table 2. Of six samples discordant with RT-PCR results, one was false negative, and five were false positive. There were three weakly positive and two positive results. The false negative sample’s Ct-values were 31.18 for *E*, 39.2 for *RdRp*, and 35.54 for *N* genes, as shown in Table 3 and Additional file 2: Supplementary Table S2.

**Table 2:** The sensitivity and specificity of the Standard Q COVID-19 Ag test

	RT-PCR assay (Allplex™ 2019-nCoV Assay)		
	Positive	^#1^Negative	Total
*Rapid SARS-CoV-2 antigen assay (Standard Q COVID-19 Ag kit)*			
Positive	59	5	64
Negative	1	389	390
Total	60	394	454
Sensitivity	98.33% (59/60; 95%CI, 91.06–99.96%)		
Specificity	98.73% (389/394; 95%CI, 97.06–99.59%)		

^#1^Negative RT-PCR is defined as having Ct-values of *E*, *RdRp*, and *N* larger than 40

**Table 3:** Cases with discordant results between the Standard Q COVID-19 Ag test and RT-PCR assays

Gender	Age	Initial diagnosis	Specimen type	Ct-value of RT-PCR			Rapid Ag test result	Interpretation
				*E*	*RdRp*	*N*		
F	33	Pneumonia	NP swab + throat swab	31.18	39.20	35.54	Negative	False negative
F	67	Pre-operative	NP swab + throat swab	> 40	> 40	> 40	Positive	False positive
M	75	Pre-operative	NP swab + throat swab	> 40	> 40	> 40	Positive	False positive
F	61	Pre-operative	NP swab + throat swab	> 40	> 40	> 40	Positive (weakly)	False positive
F	83	Pre-operative	NP swab + throat swab	> 40	> 40	> 40	Positive (weakly)	False positive
F	64	Pre-operative	NP swab + throat swab	> 40	> 40	> 40	Positive (weakly)	False positive

## Discussion

Molecular tests are the standard laboratory diagnosis to confirm SARS-CoV-2 infection; RT-PCR assays for SARS-CoV-2 RNA detection in clinical specimens are widely used in COVID-19 diagnostic laboratories. There were 183 clinical laboratories in Thailand, including our laboratory at Siriraj Hospital that passed the external quality control of RT-PCR tests by the Department of Medical Science (DMSC), Ministry of Public Health, and was authorized a COVID-19 diagnostic laboratory [15, 16]. Rapid antigen immunoassays with equivalent sensitivity and specificity to real-time RT-PCR assays will help speed up disease screening. In this study, the commercially available rapid SARS-CoV-2 antigen detection kit (Standard Q COVID-19 Ag test) was compared with the RT-PCR assay (Allplex™ 2019-nCoV Assay) for detection of SARS-CoV-2 infection.

The sensitivity and specificity of the Standard Q COVID-19 Ag test for rapid detection of SARS-CoV-2 antigen reported by the manufacturer (total n = 202; positive n = 32; negative n = 170) were 84.38% (95% CI, 67.21–94.72%) and 100.00% (95% CI, 97.85–100%), respectively. The sensitivity of this test was evaluated at a trial site in Malaysia using 32 RT-PCR-positive nasopharyngeal swabs from symptomatic patients. The specificity of this test was evaluated by the R&D team of SD Biosensor using 170 RT-PCR-negative samples. The monoclonal antibody specific to SARS-CoV-2 N antigen coated on the Standard Q COVID-19 Ag test was produced from WUHAN-01 strain, which is genetically closely related to the SARS-CoV-2 strains detected in Thailand [17, 18]. Our results showed higher sensitivity (98.33% vs. 84.38%) but less specificity (98.73% vs. 100.00%) than the manufacturer’s results. The difference in our test performance from the manufacturer could be due to various factors, including the batch of kit reagents, the sample quality and level of extracted antigen, and sample handling and processing techniques. We reduced the sample viscosity using glass beads and vortexing before adding to the extraction buffer. The filter nozzle cap provided in the kit also minimized the glutinousness of the samples. A negative test result could be due to lower levels of extracted antigen than the test’s detection limit. Our batch of clinical specimens might generally have higher viral loads (low Ct-value) than that of the manufacturer’s trial site, which enhanced the chance of antigen detection in our study.

Of 60 RT-PCR-positive samples in our study, the sole false negative result was from the NP and throat swab of a female patient with pneumonia tested for SARS-CoV-2 antigen seven days after disease onset (RT-PCR-positive case no.39). The RT-PCR result of this sample had relatively high Ct-values: 31.18 for *E* gene, 39.2 for *RdRp* gene, and 35.54 for *N* gene, which may explain the negative result of the Standard Q COVID-19 Ag test. However, the Standard Q COVID-19 Ag test correctly detected SARS-CoV-2 antigen from another female patient who also had relatively high Ct-values: 33.49 for *E* gene, 36.94 for *RdRp* gene, and 37.17 for *N* gene (RT-PCR-positive case no.23). This patient was presented with upper respiratory tract infection (URI) and was tested four days after symptom onset [see Additional file 1]. SARS-CoV-2 viral load in upper respiratory specimens was detected at a higher level soon after the symptom onset [19]; thus, a higher chance of positive antigen detection at the early phase can be implied. This SARS-CoV-2 antigen detection kit might be recommended for patients at the early time point after symptom onset where higher viral loads are anticipated. As aforementioned, some other factors such as clinical manifestation, duration from disease onset to laboratory test, type of specimens, and how the specimens were collected and processed (sample handling and processing techniques) potentially affect the result interpretation. Of 394 RT-PCR-negative samples from pre-operative cases, five NP and throat swabs were tested positive for SARS-CoV-2 antigen using the Standard Q COVID-19 Ag test. Although it is unclear what caused the discordant result, we observed that thick and highly viscous mucous tended to yield false positive results when tested with the antigen detection kit. For patients with negative SARS-CoV-2 detection by RT-PCR, clinical data (such as underlying diseases or infection with other pathogens) were not included in the study. Therefore, the possibility of cross-reactivity with other antigens cannot be excluded.

Our results showed higher sensitivity of the rapid SARS-CoV-2 antigen test (98.33% by Standard Q COVID-19 Ag test) than other rapid antigen tests previously reported. Previous studies reported a sensitivity of 93.9% (95% CI, 86.5–97.4%) by Fluorescence Immunochromatographic Assay for 2019-nCoV Ag Test (Bioeasy Biotechnology Co., Shenzhen, China), 50.0% by COVID-19 Ag Respi-Strip CORIS®, and 11.1–45.7% by BIOCREDIT COVID-19 Ag (BioVendor Research and Diagnostic Products) [10–12]. The positive and negative predictive values (PPV and NPV) of the assay could not be accurately calculated without the present population prevalence of COVID-19. However, there were five false positive samples tested by the Standard Q COVID-19 Ag test. We can estimate that in a low COVID-19 prevalence area, the PPV for this test is low. Hypothetically, in the 10% COVID-19 prevalence rate, the PPV vs NPV of the Standard Q COVID-19 Ag test would be 89.59% (95% CI, 78.27–95.37%) versus 99.81% (95% CI, 98.71–99.97%). While in the 1% COVID-19 prevalence rate, the PPV vs NPV of the Standard Q COVID-19 Ag test would be 43.91% (95% CI, 24.66–65.17%) versus 99.98% (95% CI, 99.88–100.00%). Thus, the Standard Q COVID-19 Ag test might be useful in the high prevalence area.

The advantage of the Standard Q COVID-19 Ag test as a screening for COVID-19 is its simple procedure and quick results with high NPV, but its disadvantage is low PPV in a low prevalence area. Thus, the nucleic acid test (NAT) for SARS-CoV-2 gene detection, which is more sensitive and specific than this lateral flow immunoassay, is still a standard test for COVID-19 diagnosis. Even with its limitations, the rapid SARS-CoV-2 antigen test can benefit all healthcare workers in managing infected individuals in time effectively, especially in rural and outbreak areas. Therefore, a prospective study of the rapid SARS-CoV-2 antigen test in these fields should be performed before the implementation.

## Conclusions

The rapid assay for SARS-CoV-2 antigen detection (Standard™ Q COVID-19 Ag kit) showed comparable sensitivity (98.33%; 95% CI, 91.06–99.96%) and specificity (98.73%; 95% CI, 97.06–99.59%) with real-time RT-PCR assay. We believe there is a potential use of this rapid and simple SARS-CoV-2 antigen detection test as a screening assay, especially in a high prevalence area.

## Supplementary information

**Additional file 1**. ** Table S1**: Rapid antigen test in 60 SARS-CoV-2 RT-PCR-positive cases. Characteristics of each COVID-19 Thai case (n=60) including gender, age, initial diagnosis, specimen type, Ct-value of RT-PCR (*E*, *RdRp*, *N*), RT-PCR result, Standard Q COVID-19 Ag test result, and time from symptom onset to laboratory test are demonstrated. Continuous data were presented in mean, standard deviation (SD), median, and range (min, max).

**Additional file 2**. ** Table S2**: Rapid antigen test in 394 SARS-CoV-2 RT-PCR-negative cases. Characteristics of each SARS-CoV-2 RT-PCR-negative case (n=394) including gender, initial diagnosis, specimen type, Ct-value of RT-PCR (*E*, *RdRp*, *N*), RT-PCR result, Standard Q COVID-19 Ag test result, and time from symptom onset to laboratory test are demonstrated. Continuous data were presented in mean, standard deviation (SD), median, and range (min, max).

## Data Availability

All data generated or analysed during this study are included in this published article and its additional files.

## Abbreviations

Ag: Antigen
ARDS: Acute respiratory distress syndrome
BSL: Biosafety level
COVID-19: Coronavirus disease 2019
Ct-value: Cycle threshold value
E: Envelope protein
N: Nucleocapsid protein
NAT: Nucleic acid test
NP: Nasopharyngeal
NPV: Negative predictive value
PPV: Positive predictive value
RdRp: RNA-dependent RNA polymerase
RT-PCR: Real-time reverse transcription-polymerase chain reaction
SARS-CoV-2: Severe acute respiratory syndrome coronavirus 2
